# Bacteriocins, Antimicrobial Peptides from Bacterial Origin: Overview of Their Biology and Their Impact against Multidrug-Resistant Bacteria

**DOI:** 10.3390/microorganisms8050639

**Published:** 2020-04-27

**Authors:** Alexis Simons, Kamel Alhanout, Raphaël E. Duval

**Affiliations:** 1 Université de Lorraine, CNRS, L2CM, F-54000 Nancy, France; 2 Institut Micalis, équipe Bactéries Pathogènes et Santé, Faculté de Pharmacie, Université Paris-Saclay—INRAE—AgroParisTech, 92296 Châtenay-Malabry, France; 3 ABC Platform^®^, Faculté de Pharmacie, F-54505 Vandœuvre-lès-Nancy, France

**Keywords:** bacteriocins, Gram-positive bacteria, Gram-negative bacteria, antimicrobial activities, multidrug-resistant bacteria

## Abstract

Currently, the emergence and ongoing dissemination of antimicrobial resistance among bacteria are critical health and economic issue, leading to increased rates of morbidity and mortality related to bacterial infections. Research and development for new antimicrobial agents is currently needed to overcome this problem. Among the different approaches studied, bacteriocins seem to be a promising possibility. These molecules are peptides naturally synthesized by ribosomes, produced by both Gram-positive bacteria (GPB) and Gram-negative bacteria (GNB), which will allow these bacteriocin producers to survive in highly competitive polymicrobial environment. Bacteriocins exhibit antimicrobial activity with variable spectrum depending on the peptide, which may target several bacteria. Already used in some areas such as agro-food, bacteriocins may be considered as interesting candidates for further development as antimicrobial agents used in health contexts, particularly considering the issue of antimicrobial resistance. The aim of this review is to present an updated global report on the biology of bacteriocins produced by GPB and GNB, as well as their antibacterial activity against relevant bacterial pathogens, and especially against multidrug-resistant bacteria.

## 1. Introduction

The discovery of antibiotics represents a major achievement in the management of infectious diseases, and has greatly enhanced quality of life and life expectancy all over the world. However, antimicrobial resistance (AMR) rapidly emerged a few years after the use of antibiotics, and its continuous spread has since been a major health problem [1]. Multidrug- and even pandrug-resistance to the main classes of antibiotics commonly used in clinical practice is increasingly noted for both Gram-positive bacteria (GPB) and Gram-negative bacteria (GNB) [1]. The continuously growing rate of morbidity and mortality associated with Methicillin-Resistant *Staphylococcus aureus* (MRSA), Vancomycin-Resistant Enterococci (VRE) or Multidrug-Resistant (MDR) GNB present a serious health and economic burden in both hospital and community settings, highlighting the need to develop new antibiotics [2,3,4]. It was understood that the solution comes with the rational use of already-existing antibiotics. However, in the race against microbial resistance, pressing efforts have been made in order to develop new antimicrobial agents [5].

Numerous natural resources were explored, including plants, animals and microorganisms. Combined with chemical and biotechnological tools, these resources provided several compounds that might be promising as antimicrobial agents [6,7,8]. Among natural resources, bacteria displaying the ability to antagonize other bacteria were also explored. The first bacteriocin was identified in 1925 [9], and thus enabled the development of an entire research sector composed of countless works aiming to discover new antimicrobial compounds of microbial origin in the following decades. Bacteriocins received a lot of interest as potential antimicrobial agents against different bacterial, fungal and viral species [10,11,12], and even against natural resistant structures such as bacterial biofilms [13,14]. These natural ribosomally synthetized peptides are produced by bacteria living in a competitive polymicrobial environment and are used to eliminate other bacterial species, particularly closely related ones [15,16,17]. Thus, the diversity of the different bacteriocins among bacteria provide a broad spectrum of activity [18,19,20]. The bacteriocin production by ribosomes distinguish them as Non-Ribosomally Synthesized Antibiotics (NRSAs) [21], such as lipopeptides and glycopeptides [21]. Although bacteriocins are produced by both GPB [22] and GNB [23], the vast majority of bacteriocins reported are produced by GPB, and particularly Lactic Acid Bacteria (LAB) [11,24]. These microbial compounds are widespread among bacteria species, and some studies suggest that virtually all bacteria are able to produce bacteriocins [25,26]. Due to this high diversity of producing bacteria, a large variety of bacteriocins have been identified, and some bacteria can produce several kind of bacteriocins [27]. This wide range of antimicrobial molecules allows a broad range of biotechnological, industrial and pharmaceutical applications [20,28].

Thus, one of the main sectors impacted by the use of bacteriocins is the agro-food [29]. Characterized by their probiotic nature, some LABs and their metabolic products are “Generally Considered AS Safe” (GRAS, Grade One) for the food industry [30]. Thus, LABs are used in several processes (e.g., fermentation, food preservation) due to their bio-preservative ability to inhibit competitive flora [27], and, in particular, food-borne pathogens (i.e., *Listeria monocytogenes*, *Clostridium* sp., *Staphylococcus* sp. and *Escherichia* sp.) [31,32,33]. As bacteriocin are easily degraded by proteolytic enzymes, such as proteases of the mammalian gastrointestinal tract, they may be considered as not harmful for human use [30]. In addition, unlike antibiotics, specific pathogens can be targeted, due to the narrow spectrum of certain bacteriocins without impact on commensal microflora [34]. Only one bacteriocin, the nisin, is currently approved as food preservative by the European Union (i.e., nisin was registered with the E number E234 as a food additive), the World Health Organization and the Food and Drug Administration [35]. However, the development of bacteriocins as biopreservative agents retains very strong interest. These molecules have been studied in several works related to the food industry, demonstrating, for example, an increase in the biopreservation of vegetable foods [36] or incorporated in active packaging [37]. Different ways may be used to incorporate bacteriocins in food products: (i) a direct inoculation of LAB into food products, which will produce bacteriocins, (ii) an addition of purified bacteriocin as a preservative agent or (iii) the addition as ingredient of a fermented product containing bacteriocin producers [38,39].

Another major possible utilization of bacteriocins is the fight against antibiotic-resistant bacteria [11,40]. The increase in MDR bacteria is a major concern for public health [41,42], causing a decrease in the efficiency of conventional antibiotics, and the development of a new alternative to antibiotics is a real emergency [5,43,44]. In this context, as bacteriocins are considered as weapon used by bacteria to survive, then their use to overcome drug-resistance seems to be a very interesting approach [17]. MDR pathogens such as MRSA, VRE, penicillin-resistant *Streptococcus pneumoniae* and MDR GNB (e.g., *Pseudomonas aeruginosa*, *E. coli*…) in particular retain more attention for their potential pathogenicity [11,17]. The diversity of molecules, specificity of antimicrobial mechanism, and potential synergy with other drugs are different advantages that make bacteriocins relevant in pharmacology, with some drawbacks such as the susceptibility to proteolytic enzymes and their eventual toxicity for mammalian cells [45]. Indeed, these molecules present a great potential to substitute other antimicrobial compounds or to be combined with antibiotics [11], and in vivo studies have shown their potential as a therapeutic agent in case of recurrent infections (*Mycobacterium* spp., *Streptococcus pyogenes*, etc.) [11].

This review represents an effort to highlight the antimicrobial potential of bacteriocins as possible antibiotic candidates to fight antimicrobial resistance. To better understand the mechanisms associated with an antimicrobial effect, the biology of bacteriocins (i.e., biosynthesis, transport, self-immunity) produced by both GPB and GNB is presented, as well as their mechanisms of action on bacteria. In addition, the problem of the development of resistance to bacteriocins, which may represent a potential issue for their use in future, is addressed. Finally, an overview of their antimicrobial activity against pathogenic bacterial strains demonstrates their potential as an alternative or support to conventional antibiotics. 

## 2. Classification of Bacteriocins

**Figure 1:**
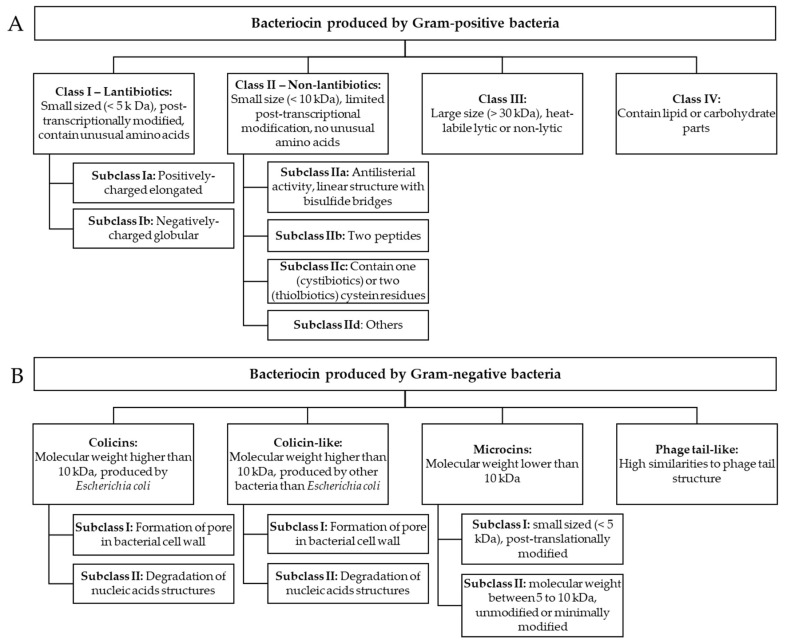
Classification of bacteriocins produced by Gram-positive (**A**) and Gram-negative (**B**) bacteria.

**Figure 2:**
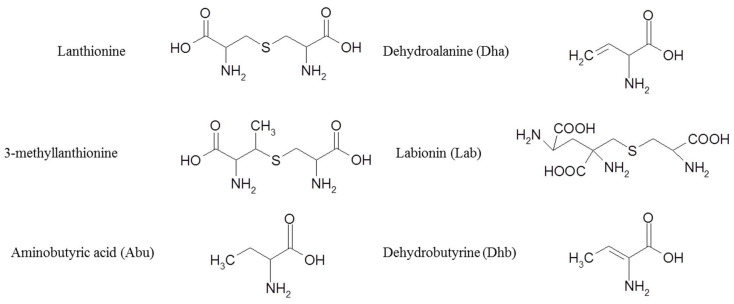
Structures of some unusual amino acids retrieved in bacteriocins produced by Gram-positive bacteria.

To date, the classification of bacteriocins was an issue due to the important variety in structure and activity. Different classification criteria were employed by authors, which could be confusing for the reader. Indeed, attempting to regroup all known bacteriocins in distinct groups is nearly impossible due to the huge number of bacteriocins currently reported and the overlapping structural, functional and genetic characters they present. Databases such as APD3 have been developed to list antimicrobial peptides [46], and recent works have regrouped bacteriocins into different classes, depending on several factors such as their size, molecular composition and structure or modification process [19,30,32] (Figure 1, Figure 2). The first differentiation between bacteriocins that can be done is the type of the producing organism: GPB or GNB.

### 2.1. Bacteriocins Produced by Gram-Positive Bacteria (BGPB)

BGPBs are currently classed into four different classes [30,32]:

Class I is also named lantibiotics and includes small sized (<5 kDa) and post-transcriptionally modified bacteriocins. A common feature of this group is the presence of unusual amino acids such as dehydrated amino acids, lanthionine and 3-methyllanthionine forming multiple ring structures and conferring structural stability to heat, pH and proteolysis (Figure 2) [47]. The presence of these amino acids is the result of post-translational modifications consisting of the dehydration and cyclization of specific amino acids residues. This class is usually associated with an inhibition of GPB and food-borne pathogens [48]. Lantibiotics may be further classified into subclasses. Positively charged elongated peptides are categorized in subclass Ia (nisin, epidermin, gallidermin, etc.) (Figure 2) [49], and are usually associated with the formation of the pore into bacterial membranes. Concerning subclass Ib, the structure of these bacteriocins is globular and inflexible, and these peptides are negatively charged (e.g., lacticin 481, cytolysin, salivaricin). The mechanism of action of this subclass is related to the inhibition of specific enzymes which are essential for the targeted bacteria.

On the other hand, class II, or non-lantibiotics, do not contain unusual amino acids in their structure [22] and post-translational modification is limited to bisulfide bridge formation in only a few members (e.g., pediocin PA-1, pediocin AcH) (Figure 1). As class I bacteriocins, these peptides are also heat-stable and small in size (<10 kDa), and mainly induce a destabilization and permeabilization of the bacterial membranes or cause pore formation into the membrane [50,51]. This group can be divided into four subclasses. The subclass IIa members exhibit a linear structure showing bisulfide bridges and a common antilisterial activity thus referred to as antilisterial bacteriocins (e.g., leucocin A, acidocin A, pediocin PA-1) [52,53]. Subclass IIb bacteriocins are two-peptide bacteriocins (α/β) that are equally produced and both necessary to exhibit antibiotic activity (e.g., lactococcin G, lactococcin Q and plantaricin NC8) [54]. Subclass IIc are small bacteriocins associated with a leader peptide sequence and may include one to two cysteine residues in their structure (named, respectively, cystibiotics and thiolbiotics). This subgroup includes several molecules such as lactococcin A, divergicin A or acidocin B [51]. Finally, the subclass IId is used to gather all bacteriocins included in the Class II group which were not included in the different subgroups presented above.

Contrary to class I and class II bacteriocins, class III bacteriocins are large peptides (>30 kDa), and may be heat-labile lytic or non-lytic [55,56]. Bacteriocins such as zoocin A, lysotaphin or helveticin J and V are included in this group [55]. These bacteriocins have an antibacterial activity linked to enzymatic activity (e.g., endopeptidase), leading to the disruption of the bacterial cell wall. 

Class IV bacteriocins are specified by their structure, containing lipid or carbohydrate parts [48], such as plantaricin S [57] or leuconocin S, which disrupt bacterial cell membrane. This structural particularity makes these molecules sensitive to several enzymes (i.e., glycolytic or lipolytic enzymes).

### 2.2. Bacteriocins Produced by Gram-Negative Bacteria (BGNB)

The narrower spectrum of antimicrobial activity of BGNB limits their use compared to BGPB [28]. However, this category of bacteriocins still represents an important part of the antimicrobial peptides. Among the bacteriocins produced by GNB, most of them were isolated from *Escherichia coli* strains, but several other genera such as *Pseudomonas* or *Klebsiella* may also produce antimicrobial peptides. BGNB are divided into four different categories [23,58] (Figure 1B):

The first group is the colicins, which are the bacteriocins that have a molecular weight higher than 10 kDa and are produced by *E. coli*. These peptides have been used for decades as models for the bacteriocin structure and the study of their functional evolution. The mechanism of action of colicins can be distinguish into two types [15,59,60]: (i) the formation of pores in the bacterial cell wall (i.e., colicins A, B, E1, Ia, Ib, K and 5) and (ii) the degradation of nucleic acid structures similar to DNAses, RNAses or tRNases (i.e., colicins E2 to E9).

The second group gathers Colicin-like bacteriocins, which are produced by other bacteria (e.g., *Klebsiella* spp.: klebicins; *P. aeruginosa*: S-pyocins) ut are still similar in structure, size and function to the bacteriocins produced by *E. coli*. As colicins, their antimicrobial action can be due to pore-formation or nuclease activity [61].

The microcins represent the third group and bring together small peptides (<10 kDa) [62]. Two subclasses may be defined: (i) Subclass I are post-translationally modified bacteriocins, with a molecular weight lower than 5 kDa (e.g., microcins B17, C7, J25, D93), and (ii) Subclass II are unmodified or minimally modified peptides, and present higher molecular weight ranging from 5 to 10 kDa (e.g., microcins E492, V, L H47, 24). Microcins interact with several and diverse cellular targets, leading to various modes of action such as disruption to the membrane (e.g., microcin E492) or the inhibition of vital enzymatic functions like the ATP synthase complex (e.g., microcins M, H47), the RNA polymerase (e.g., microcin J25), the DNA gyrase (e.g., microcin B17) or the aspartyl-t RNA synthetase (e.g., microcin C).

Certain high-molecular-weight peptides present cylindrical structures that are able to perforate bacterial cell membrane and then lead to cell death [63,64]. These structures are highly similar to phage tail structure, so these antimicrobial peptides are named Phage Tail-Like bacteriocins. They form the fourth group. These peptides come from the domestication of phage tail genes (i.e., the production of a needle-shaped protein structure, genes implied in the peptide release, regulatory genes). The most studied bacteriocins of this group are R- and F-pyocins produced by *P. aeruginosa* and cause a disruption of the membrane potential leading to the formation of pores in the bacterial membrane [61]. 

Although microcins and colicins are produced by the same species and possibly by the same strain, differences regarding their spectrum of antibacterial activity and the consequences of their secretion are notable [59]. Microcins exhibit a larger antibacterial spectrum that may cover some GNBs other than *E. coli* [62]. Moreover, their secretion is not lethal to the producer cell. On the other hand, colicins and colicin-like are produced by some GNB that have a specific bactericidal effect against sensitive strains of the same producer species [15]. In addition, the production of colicins is lethal to the producer strain as their release is associated with membrane lysis and cell death. Some significant differences are also observed among colicins and colicin-like. For example, the production of colicin is related to a gene cluster composed of three related genes in close proximity, while the lysis gene is absent for pyocin S3 [65].

## 3. Bacteriocin Biosynthesis

### 3.1. Biosynthesis of BGPB

**Figure 3:**
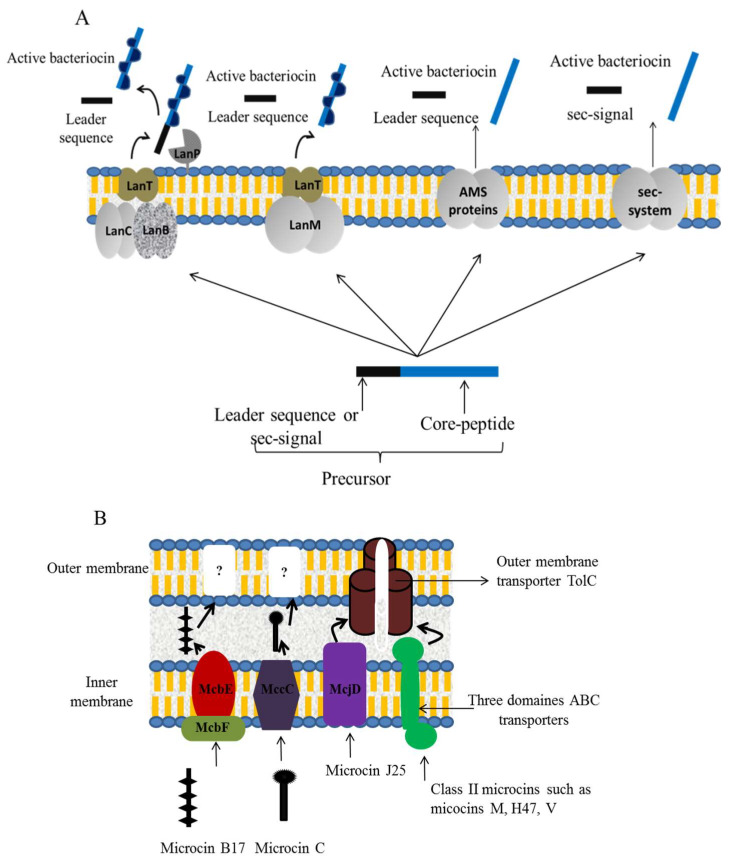
Transport mechanisms involved in the export of BGPB (**A**) and BPGN (**B**) from the producer strain.

Plasmids or chromosomes can carry genetic elements of BGPB [22], which are usually associated with transferable elements [11]. Several genes are involved in the production of class I BGPB (e.g., nisin) and are generally assembled as clusters containing structural, regulatory, modification, transport and self-immunity genes [22]. No specialized post-translational genes are involved in class II BGPB and maturation generally occurs concomitantly with transport (Figure 3A) [47]. Some BGPBs are synthesized by ribosomes, such as pre-peptides composed of an N-terminal leader peptide and a C-terminal pro-peptide [47]. The leader peptide may be used as a protector for the producer strain from its own bacteriocin, since it keeps the bacteriocin inactive as long as it is not secreted [22,66]. In addition, it was noted that the leader peptide plays a crucial role in the maturation of class I BGPB [66]. Some class II BGPB contain a sec-dependent N-terminal leader peptide that is necessary for their transport via the general secretory sec-pathway [47]. Concerning certain class I bacteriocins, the pro-peptide is subjected to post-translational modifications to be transported via an ABC transporter to an extracellular space where a serine protease cuts the leader peptide and releases the mature lantibiotic [49]. In other cases, transport of the formed pro-peptide and cleavage of the leader peptide is concomitant and performed by one proteolytic enzyme belonging to the ABC transporters family [47]. Subclasses IIa and IIb BGPB may use specialized ABC transporter maturation and secretion (AMS) proteins that concomitantly transport and cleave the leader peptide or be transport via the sec-dependent pathway due to the presence of a sec-signal peptide in their pre-peptide structure [47].

To date, stress-inducible production of BGPB has not been documented, and the production of BGPB appears to be constitutive and auto-regulated [22,67]. Depending on the BGPB, the mechanism of auto-induction is not clarified with the same depth as demonstrated for the well-known nisin. Indeed, it was revealed that the regulatory system of nisin production is a two-component system composed of a membrane-bound sensor, a Histidine Protein Kinase (HPK), which detects an extracellular signal, and a cytoplasmic Response Regulator (RR) that can induce the expression of nisin structural gene [49]. It was reported that modified nisin, mutant nisin species and nisin analogues still induce the transcription of the *nisA* structural gene by acting as extracellular signals to the HPK [49]. In a similar way, the regulation of epidermin biosynthesis was reported to be mediated via the protein EpiQ, which presents some similarities with RRs [68]. 

### 3.2. Biosynthesis of BGNB

Biosynthesis pathways of bacteriocins produced by GNB may differ depending on the producing organism. Microcins are generally overproduced in stress conditions such as starving and the stationary phase of bacterial growth [62]. Genetic determinants for microcin production are either plasmid or chromosome borne and organized in clusters including structural genes, self-immunity genes, export genes and post-translational modification genes for subclass I microcins [62]. Microcins synthesis is initiated with ribosomally synthetized pre-peptides containing an N-terminal leader sequence that has to be cleaved in order to be activated [69]. The leader peptide was reported to be necessary for microcin intracellular stabilization and to play the role of a folding chaperone, enabling the molecule to be recognized by the export system [70]. Moreover, for subclass I microcins, the leader peptide was noted to be used for recognition by enzymes mediating the post-translational modification of these peptides [62]. Cleavage of the leader peptide seems to be mediated by the export system during microcin translocation. The processing of microcin C still represents an exception to this mechanism since it is not performed in the producer strain but in the target strain [71]. Export mechanisms of some microcins from the producer strains need further elucidation. The export of microcin B17 was reported to be ensured by ABC transporter-related proteins, MccE and MccF, acting cooperatively as an efflux pump transporting microcin B17 to the periplasmic space (Figure 3B) [71]. For microcin C, transport is thought to be performed by a hydrophobic protein resembling the multidrug efflux transporters involved in the export of small solutes, such as sugars and secondary metabolic products (Figure 3B) [72]. For both bacteriocins, the mechanism of transport across the outer membrane was not elucidated. The passage of microcin J25 across the inner membrane was reported to be mediated by McjD, a protein belonging to the family of ABC transporters [73], and cooperates with an outer membrane trimeric protein, TolC, that forms a channel leading microcin J25 outside the producer bacteria (Figure 3B) [74]. Similarly, subclass II microcins employ special three domains ABC transporters to cross to the inter-membrane space and the TolC outer membrane transporter to be exported outside the producer strain (Figure 3B) [62,70]. These ABC transporters exhibit a protease activity in their cytosolic domain and communicate with TolC via their periplasmic domain [70].

Concerning colicin production, genetic elements are plasmid-borne and much simpler than those involved in microcins or BGPB production. Indeed, only one to three genes can be retrieved in colicin operon including the structural gene, immunity gene and lysis gene [15]. Each colicin operon can be induced by stress signals due to a specific SOS promoter that regulates its transcription [59], and various stress factors were identified as colicin inducers (e.g., nutritional limitation, oxygen starvation, DNA damage, stationary phase of growth). In this context, the production of colicins itself might be considered as an SOS response, since their transcription is regulated by LexA protein, the repressor of SOS genes [59,75]. Colicins are synthesized without any post-translational modification and their peptide sequence contains three functional domains common to all colicins: i) a N-terminal translocation domain, ii) a central receptor-binding domain and iii) a C-terminal cytotoxic domain [76,77]. Colicins are secreted in non-specific mechanism consisting of bacterial membrane lysis by dedicated lysis factors [15].

As examples of colicin-like and phage tail-like bacteriocins, pyocins produced by *P. aeruginosa* might present a particular interest due to their activities against relevant Gram-negative pathogens [61,78]. Three types of chromosome-encoded pyocins could be found in *P. aeruginosa*, including R, F, and S-type pyocins [15]. R- and F- types look like tails of bacteriophages, and R-type pyocins have a non-flexible and contractile structure while the F-type has a flexible and non-contractile structure [65]. They are composed of two tightly bound peptides, including the killing peptide and the immunity peptide [79]. The killing domains of S-type demonstrate a close evolutionary relationship with several colicins [79]. Like colicins, pyocins are inducible by DNA mutagenesis and present a single hit-killing mechanism [61]. 

## 4. Mechanism of Action

### 4.1. Antimicrobial Mechanisms of Bacteriocins

**Figure 4:**
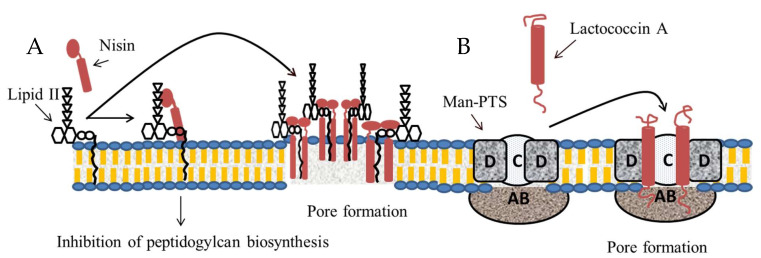
Mechanisms of action of nisin and lactococcin A. (**A**) By targeting lipid II, nisin can inhibit peptidoglycan biosynthesis and form pores in the bacterial membrane. (**B**) Lactococcin A uses the mannose phosphotransferase system (Man-PTS) as a receptor, leading to uncontrolled opening of this receptor and thereby forming a pore in the bacterial membrane.

BGPB antimicrobial action is usually associated with a disruption of the bacterial membrane integrity, leading to cell death [80]. Among the various possible mechanisms implied, this effect can be the result of direct interaction with the lipid II component of bacterial membrane, the Mannose PhosphoTransferase System (Man-PTS) or without a specific receptor being involved [47]. Nisins are reported to act by pore formation using lipid II as docking molecule, leading to increased membrane permeability of the targeted cell, and thereby its death (Figure 4A) [81]. The nisin peptide structure can be divided into two functionally distinct domains: i) the N-terminal domain that contains two rings presenting a high affinity to pyrophosphate groups of lipid II; ii) the C-terminal domain, which is essential for pore formation [82]. Similar findings were also reported for other subclass Ia BGPBs such as epidermin and gallidermin. On the other hand, pore formation by lacticin 3147, a two-peptide lantibiotic (LtnA1 and LtnA2), is performed at first by the LtnA1 association with lipid II and then the complex lipid II-LtnA1 is able to recruit LtnA2, which enters the membrane and forms a pore [83]. As for microbisporicin, other bacteriocins can have an antimicrobial effect based on an enzymatic inhibition of peptidoglycan biosynthesis, leading to a cytoplasmic accumulation of peptidoglycan precursors and a disruption of bacterial membrane [84]. Various class II bacteriocins (e.g., pediocin PA-1, sakacin P, lactococcin A) targets as receptor Man-PTS [47], a transport system used to couple import and for the phosphorylation of sugars [85] (Figure 4B). The enzyme EII present in this system, which is a carbohydrate-protein-specific complex, is composed of three proteins (i.e., AB, C, and D) and represents the target of these bacteriocins [86]. The interaction of bacteriocin with the Man-PTS results in a permanent opening of this receptor, and thus an uncontrolled and continuous efflux of intracellular molecules. Subclass IIb bacteriocins such as the two-peptide bacteriocins lactococcin Q and lactococcin G also act by pore formation, probably by interacting with membrane proteins as receptor [47].

**Figure 5:**
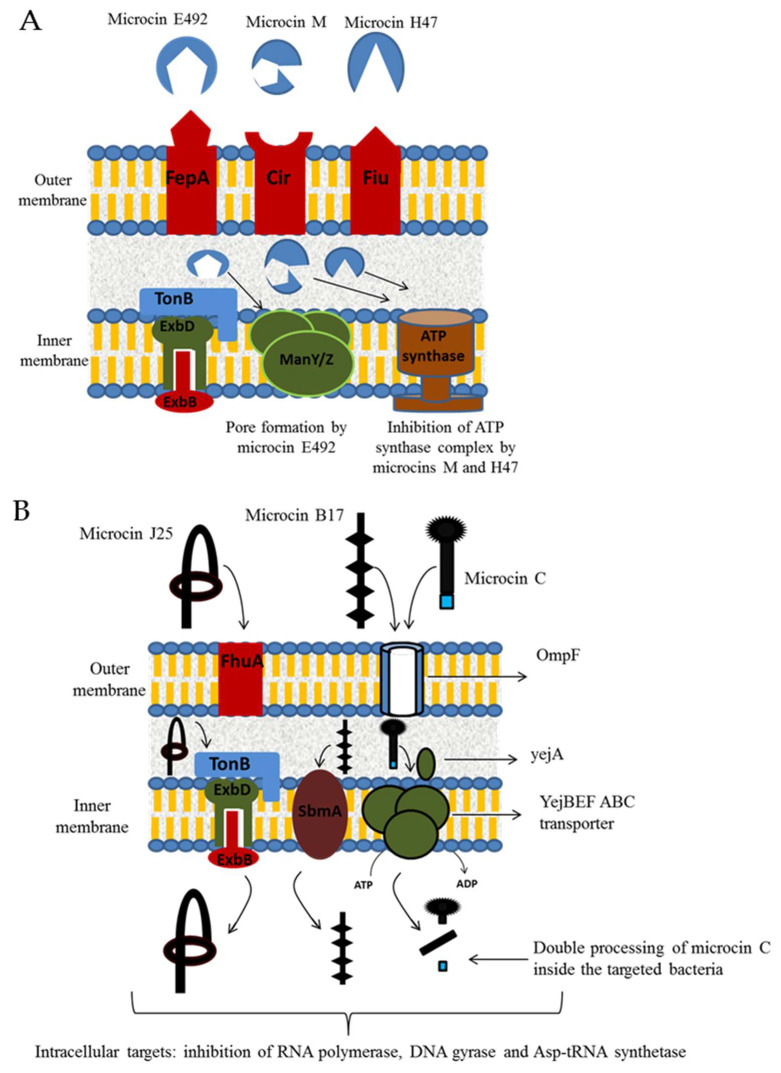
Mechanisms of microcins’ entry inside sensitive bacterial strains and their inner membrane (**A**) and intracellular (**B**) targets.

**Figure 6:**
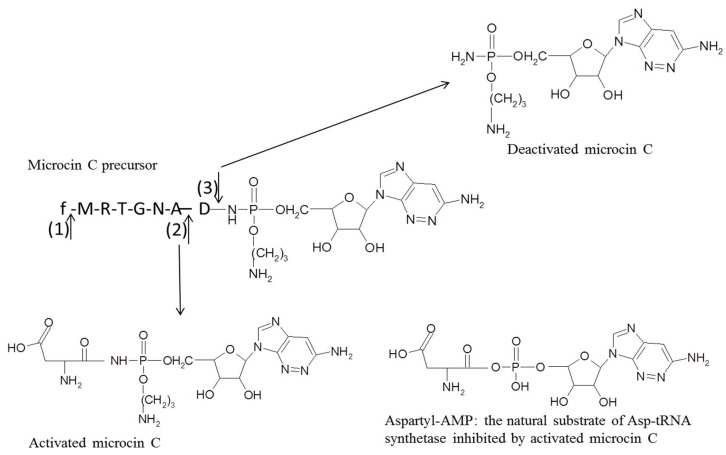
Structure of microcin C precursor and site of cleavage by deformylase (1), aminopeptidases enzymes (2) and serine proteases (3). Microcin C is double processed in bacterial targeted cells, first by deformylase and then by specific aminopeptidase, releasing the active microcin C which is an aspartyl-AMP analogue that competes with this natural substrate and inhibits tRNA-synthetase in sensitive strains. Specific serine proteases can deactivate microcin C in some resistant strains.

Concerning BGNB bacteriocins, microcins exhibit their antibacterial activity via one of two mechanisms: (i) pore formation in the inner membrane (e.g., microcins E492, M and H47); (ii) targeting of intracellular enzymes (e.g., microcin J25, B17 and C) (Figure 5) [62]. Unlike BGPB, microcins must enter the targeted cell to have an antimicrobial activity, and then use specific receptors at the outer membrane of the sensitive strains, including receptors involved in iron uptake and outer membrane porins (Figure 5) [62,87].Thus, the siderophore transport system is used for importing pore-forming microcins [62]. Indeed, iron uptake receptors such as FepA, Cir, Fiu or FhuA may be targeted and act as a receptor for several microcins, respectively microcin E492, M, H47 or J25 [88,89]. Pore-forming by microcins is due to a subsequent interaction with specific components in the inner membrane, such as those implicated in the absorption of mannose and related hexoses (Man XYZ permease) for microcin E492, and the ATP synthase complex for microcin H47, while the target of microcin M remains unidentified (Figure 5A) [90,91]. Concerning microcins targeting intracellular enzymes, uptake may be mediated by the siderophores transport system such as for microcin J25 (Figure 5B) [87]. On the other hand, microcin C and microcin B17 use the outer membrane protein OmpF as a channel to cross to the periplasmic space of targeted cells (Figure 5B) [71,87]. To enter inside the cell, microcin B17 employs specific inner membrane receptors including SdaC and SbmA proteins that are required for nutriment uptake (Figure 5B) [87]. Microcin C was reported to be transported through the inner membrane by the YejABEF ABC transporter complex (Figure 5B). Once inside the cell, these microcins inhibit specific and essential enzymes. For example, microcin J25 was noted to inhibit RNA polymerase of the targeted bacteria while microcin B17 was reported to be a DNA gyrase inhibitor, blocking DNA replication and inducing an SOS response [87]. Microcin C was reported to act by inhibiting the aspartyl-tRNA synthase and consequently blocking protein synthesis in the targeted cell [92]. After uptake by bacterial cell, microcin C is processed, and first subjected to the action peptide deformylase that cleaves the formyl group from the N-terminal of the heptapeptide (Figure 6) [71,93]. Then, the peptide sequence is cleaved by the any one of the three aminopeptidases A, B, and N, yielding a non-hydrolyzable aspartyl adenylate analog that competitively inhibits the enzyme aspartyl-tRNA synthetase (Figure 6) [71].

**Figure 7:**
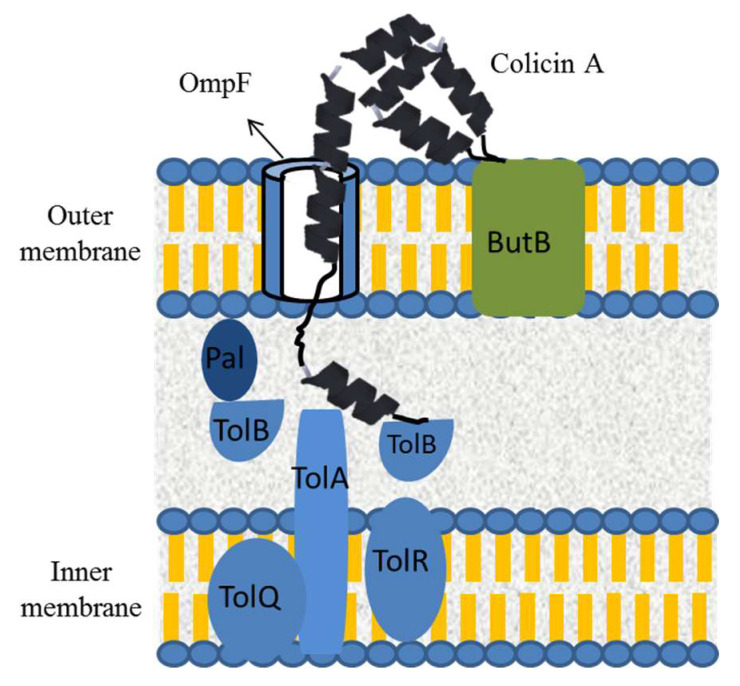
Colicin A entry inside sensitive bacterial strains. Colicin A first binds to the ButB outer membrane receptor and then uses the OmpF translocator to cross the outer membrane. Colicin A is then guided by the TolA-Pal system and its pore-forming domain is inserted into the inner membrane.

As for microcins, colicins share general mechanistic aspects and act by pore formation in sensitive bacterial membrane or by intracellular enzymatic degradation of specific targets [59]. These bacteriocins also use specific receptors in the outer membrane in order to enter inside the targeted cells [15]. Several colicin receptors have been described, such as the TonB-dependent vitamin B12 transporter (BtuB), the outer membrane proteins A and F (OmpA and OmpF), the nucleoside transporter Tsx and receptors involved in iron uptake such as FepA, FhuA, and Cir [15]. Subclass I colicins generally interact with two outer membrane receptors, first with BtuB, for colicin A and E, for example (Figure 7), or Tsx for colicin K, and then with an OmpF acting as translocator [94,95,96]. Translocation by OmpF is dependent on the Tol-Pal system present in the periplasm of targeted bacteria, as exemplified for colicin A in Figure 7 [97]. On the other hand, subclass II colicins, such as colicin B and D, interact with a single receptor generally involved in iron and other nutrient uptake and are translocated across the inner membrane by the TonB system consisting of TonB, ExbB and ExbD in a way that may resemble that observed for some microcins [94]. Pore-forming colicins such as colicin A, K and N act by depolarizing the cytoplasmic membrane while those having enzymatic activities catalyse the degradation of essential compounds for bacterial survival such as DNA, tRNA or rRNA [15]. The mechanism of action of colicin M is unique and underlays the inhibition of peptidoglycan biosynthesis in the periplasmic space [98]. For phage tail-like bacteriocins, the mechanism of antibacterial action of R- and F-pyocins was reported to be mediated by membrane depolarization resulting from pore formation in the targeted cell [99]. Interestingly, colicin-like pyocins such as the S-type showed various mechanisms of action that can also be encountered in colicins including DNase, tRNase and pore-forming activities [61].

### 4.2. Self-Immunity Mechanisms

**Figure 8:**
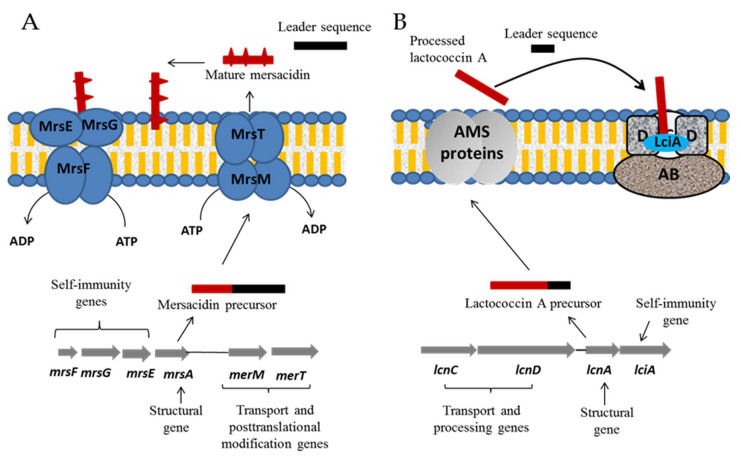
Self- immunity and transport mechanisms of mersacidin (**A**) and lactoccocin A (**B**) that target lipid II and Man-PTS, respectively. Proteins employed for self-immunity and transport of these bacteriocins are distinct and encoded by different genetic elements, as presented.

Bacteriocin production can be lethal to the producer strain if specific protection mechanisms are not employed, leading to the employment of self-immunity mechanisms [62,100]. As presented above, BGPB antibacterial activity is related by targeting lipid II component or Man-PTS in bacterial membrane [47]. Protection mechanisms of lipid II-targeting BGPB involve an ABC transport system as well as specific self-immunity proteins [101,102]. Thus, membrane-bound BGPB are rapidly removed from the membrane by the ABC transporter system in order to protect the producer strain from being killed by its own bacteriocin [101]. It should be noted that the ABC transporter used for self-immunity is distinct from that involved in bacteriocin transport outside the cell and encoded by different genes [103,104]. For example, in the case of the production of mersacidin by *Bacillus amyloliquefaciens*, the mersacidin precursor MrsA is modified by MrsM, producing a tetracyclic structure, and then processed and exported by MrsT as a mature mersacidin (Figure 8A) [102]. In order to ensure self-immunity, another ABC transporter is coded by an operon containing three genes, *mrsE*, *mrsF* and *mrsG* [103,105]. In the end, for lantibiotics (e.g., mersacidin) immunity results from the combined action of a cognate immunity protein, which binds to bacteriocin molecules on the bacterial membrane, and a multi-component ATP-binding cassette (ABC) transporter, which removes the bacteriocin from the cells [106]. In addition, transmembrane proteins involved in self-immunity may be encountered for some BGPB. By cooperating with the ABC transporter, these proteins bind the bacteriocin and help to prevent its lethal effect against the producer strain, as described for nisin [107]. The self-immunity of Man-PTS-targeting BGPB (e.g., lactococcin A) is mediated by specialized proteins that tightly bind the bacteriocin, preventing it from affecting the function of the Man-PTS of the producer strain (Figure 8B) [104]. For subclass IIb BGPB, self-immunity is not clearly understood, but it was suggested that a specific protein is involved in the protection of producer strain by interacting with the bacteriocin receptor [47].

Currently, it remains unclear how producer strains can be immune to their own microcins. Proteins mediating the export of subclass I microcins may be a way to clear out the lethal peptide and then be a kind of self-immunity mechanism [62]. In the case of microcin C, proteins acting as an efflux pump were suggested to play a role in immunity to this microcin [62]. However, it was recently shown that a specific serine peptidase is also involved in self-immunity [108]. This enzyme was reported to deactivate intact microcin C by cleaving an amide bond linking the peptidyl moieties to the nucleotide part, and was also described to confer resistance to microcin C in non-producer species (Figure 6) [108]. For some class II microcins, dedicated self-immunity proteins were identified and presented as two to three transmembrane proteins able to bind tightly to the microcin, preventing its interaction with the membrane [62]. Concerning the colicin group, self-immunity proteins of enzymatic colicins bind tightly to the catalytic C-terminal domain of these colicins and either block the active site of the enzyme or its substrate-binding site [76]. Immunity proteins of pore-forming colicins are present in the inner bacterial membrane and act by binding the cognate colicin and preventing channel formation in the inner membrane of the producer strain [109]. These proteins are noted to have a strong affinity to the corresponding colicins, protecting the producer at 104 to 107 times the concentration of colicin that would kill a non-immune cell [15]. Finally, self-immunity for phage tail-like bacteriocins such as R- and F-type pyocins is ensured thanks to the absence of a related specific receptor, which is present in sensitive cells [110].

### 4.3. Mechanisms of Bacteriocin Resistance

As for antibiotics, acquired resistance may appear through the use of bacteriocins [106], even if this phenomenon appeared to be minimized in comparison to antibiotics [32]. Resistance is reported to be a complex process, which ultimately leads to modifying membrane structure, fluidity and charge. Interaction with bacterial membrane is a prerequisite for almost all bacteriocins in order to exhibit their antibacterial effects. Thus, such modification is expected to influence bacteriocin activities [82]. As an example, resistance to nisin in *L. monocytogenes* was reported to be associated with the two-component system, Vir R/Vir S, involved in *L. monocytogenes* virulence [111,112]. This two-component system regulates the expression of *dltA* and *mprF* genes that encode specific membrane modifications involving the addition of hydrophobic components to the bacterial cell surface [112]. These modifications result in a reduced negative charge of the bacterial membrane, which repulses cationic peptides such as bacteriocins. In addition, membrane modifications leading to increased thickness and rigidity of bacterial membranes were reported to be associated with nisin resistance [113]. Moreover, the production of a nisin-degrading enzyme, nisinase, was noted to occur in some nisin-resistant GPB [111]. Resistance to subclass II was reported to be associated with down-regulated Man-PTS gene expression in *L. monocytogenes* and *Lactococcus lactis*, which was also associated with a shift in sugar metabolism from mannose or glucose to galactose [114]. In other resistant species, normal expression of this gene was noticed, indicating that a different mechanism of resistance might be involved (i.e., modification of membrane composition) [111,114]. It was also shown that resistance to various lantibiotics could be noticed in some *Clostridioides difficile* strains and was associated with the over-regulation of a non-contiguous two-component system composed of the CprK sensor kinase and an orphan response regulator, CprP [115]. This system activates the transcription of an ABC transporter system showing homology to ABC transporters involved in self-immunity to BPGB [115,116]. 

Resistance to microcins is described in some bacterial species and is generally mediated by microcin degradation, efflux pump or modification microcin intracellular targets [62]. The resistance of *Bacillus anthracis* to microcin C was shown to be mediated by a serine protease enzyme, MccF, analogue to that used in the self-immunity of the producer stains (Figure 6) [108]. This enzyme mediates the cleavage of an amide bond between the C-terminal aspartate and the nucleotide component of activated microcin C, abolishing its ability to inhibit tRNA synthetase [108]. Intrinsic resistance to microcin J25 was observed in some *E. coli* strains and demonstrated to be due to the action of a YojI protein, an ABC exporter localized in the inner membrane which is capable of pumping out microcin J25 [117]. However, this exporter protein needs to cooperate with the multifunctional outer membrane protein TolC that acts as a channel driving the microcin out of the cell, in order to ensure full protection against microcin J25 [117]. Another component possibly involved in resistance to microcin J25 is the leucine-responsive regulatory protein (Lrp) [118], as Lrp plays an important role in nitrogen metabolism and one-carbon metabolism in bacteria, permitting adaptations to different nutritional states [119]. It was indicated that the mechanism associated with this protein is the induction of the expression of the YojI microcin exporter [118]. Further data showed that accumulation of guanosine pyrophosphate compounds in *E. coli* cell during the stationary phase of growth was linked to increased resistance to microcin J25 [120]. Indeed, guanosine pyrophosphate compounds, including guanosine tetraphosphate and guanosine pentaphosphate, are bacterial alarmones synthesized by enterobacteria during amino acid limitation periods inciting various adaptive responses [120]. It was thus demonstrated that accumulation of guanosine pyrophosphate compounds also induces the expression of the YojI microcin exporter [120]. Intrinsic resistance to microcin B17 was documented in some *E. coli* strains and was related to mutant DNA gyrase, but with an altered function of this enzyme which significantly impacts bacterial growth [121]. Another mechanism by which *E. coli* may resist microcin B17 was reported to be mediated by a cytoplasmic protein, SbmC [122]. This protein is able to recognize and sequester microcin B17 inside that targeted cell and also block microcin B17 export from the producing strains [122].

Resistance to colicin in some *E. coli* strains was reported to be associated with altered colicin receptors and/or intracellular targets [15]. Colicin-tolerant *E. coli* strains were also described to present normal colicin receptors but altered translocation machinery [15]. Siderophore over-production in some strains of *E. coli* was documented to mediate resistance to subclass II colicins by competing with colicins at corresponding outer membrane receptors [15].

## 5. Antimicrobial Activity against Human Pathogens

Nowadays, the increase in bacterial antibiotic resistance is a major health issue. Among the different ways studied to fight this problem, the use of bacteriocin alone or in conjugation with drugs represents an important field of research [17,80], resulting in the use of bacteriocins in the pharmaceutical industry [28]. Indeed, bacteriocins have an interestingly diverse spectrum of antimicrobial activity [123], depending on the peptide structure or its physiochemical properties. Some bacteriocins show a specific antibacterial activity against species that are closely related to the producers, while others may exhibit a broad antibacterial spectrum (Table 1) [22]. Thus, these peptides exhibit great potential to inhibit the growth of certain antibiotic-resistant bacteria [11,40]. However, caution must be taken because the structure of a considerable number of bacteriocins is not clearly characterized and antimicrobial activity evaluation are sometimes limited to indicator or reference strains, while data relating to resistant bacteria are limited. Moreover, the antibiotic activity of bacteriocins may use different protocols, which do not enable comparison between MIC values across studies (Table 1).

### 5.1. Antimicrobial Activity of BGPB

Although the number of BGPB reported lately is notably increasing, in particular for the inhibition of food-born microorganisms, some studies have focused on tests against MDR isolates. In this context, three BGPB (i.e., bacteriocin VJ13, bacteriocin PJ4 and paracaseicin A) were identified and showed interesting antibacterial activity against several GPBs and GNBs, including pathogenic species [149,150,151]. It should be noted that the primary structure of these compounds was not determined. However, the authors drew conclusions about their proteinaceous nature by demonstrating their sensitivity to proteases, pH and temperature, and their resistance to other enzymes such as lipase and amylase. Bacteriocin VJ13 was isolated from *Pediococcus pentosaceus* and demonstrated to have a broad antibacterial activity against *L. monocytogenes*, *S. aureus*, *Clostridium* sp., *Klebsiella pneumoniae* and other GPB and GNB (Table 1) [151]. Moreover, the antilisterial activity of the isolated bacteriocin was not affected by lipase, with a pH ranging from 2 to 8 or temperature (100 °C) [151]. Bacteriocin PJ4, produced by *Lactobacillus helveticus*, was reported to be active against a panel of Gram-positive and Gram-negative pathogens such as *Enterococcus faecalis*, *S. aureus*, *E. coli*, *P. aeruginosa*… (Table 1) [150]. Bacteriocin PJ4 was relatively heat-stable and retained full activity in a pH range of 2-6, while proteolytic enzymes completely abolished its activity [150]. Paracaseicin A was isolated form *Lactobacillus paracasei* and was reported to be active against various MDR GPB and GNB (Table 1) [149]. This bacteriocin showed stable antibacterial activity after treatment with trypsin, lipase and temperatures from 60 to 120 °C, while its activity markedly diminished when treated with pepsin and α-chymotrypsin [149]. This bacteriocin also conserved its antibacterial activity in a pH range of 2-5 but was completely inhibited in physiological pH [149]. Among structurally characterized BGPG, microbisporicin, a lantibiotic isolated from the *Microbispora corallina*, was described as two similarly active and structurally related peptides (A1 and A2) exhibiting a broad-spectrum activity against various MDR GPB and some GNB (Table 1) [84]. The in vivo activity of this bacteriocin was further tested in animal models of severe infection [84,144]. Microbisporicin (NAI-107) was administered intravenously to rats infected with a penicillin-intermediate *S. pneumoniae*, MRSA, Glycopeptide-Intermediate *S. aureus* (GISA) and VRE strains. In this study, NAI-107 displayed comparable activity to reference treatments such as linezolid or vancomycin that were also tested in the same study [144]. Dose-proportional bactericidal activities were generally observed for NAI-107 in all studied infection models. Using granuloma pouch model, a single 40-mg/kg dose was reported to cause a 3-log10 (CFU/ml) reduction of viable MRSA in exudates that persisted for more than 72h [144]. Moreover, in rat endocarditis induced by MRSA, dosing regimens of 5, 10, or 20 mg/kg/day were tested indicating that NAI-107 could reduce bacterial load in heart vegetations in a dose-dependent mode [144]. Bacteriocins possessing specific anti-clostridium effect were described such as exemplified by the bacteriocin NVB302 which is isolated from *Actinoplanes liguriae* [155]. This lantibiotic demonstrated selective in vitro activity against *C. difficile* that was also comparable to vancomycin as demonstrated using in vivo gut model of *C. difficile* infection [155]. In addition to natural antimicrobial activity, the modification of the structure of certain bacteriocins may enhance their activity against antibiotic resistant strains, as shown for modified bottromycin A2 against MRSA and VRE [156].

Several other lantibiotics such as nisin, planisporicin, Pep5, epidermin, gallidermin, mutacin B-Ny266, lacticin 3147, actagardine have also shown in vitro activity against clinically important Gram-positive pathogens (i.e., *S. pneumoniae*, Staphylococci, VRE, *Propionibacterium acnes* and *C. difficile*) [157]. Bacteriocins can also be associated to other drugs in order to enhance or restore their antimicrobial activity. Thus, the use of nisin in addition to polymyxin E and clarithromycin results to a synergic effect against *P. aeruginosa* [158]. In the same way, the use of nisin, ramoplanin and other non β-lactam antibiotics shown an increase in the effect against several MRSA and VRE strains [158,159].

Another feature highlighting the antibacterial potential of bacteriocins is their activity against bacteria growth as biofilm. Indeed, biofilm formation represents an effective resistance mechanism by which bacteria protect themselves from host defenses and antimicrobial agents [133]. Infections involving biofilm formation represent a critical problem, especially for cystic fibrosis patients and patients in intensive care units developing device-related infections that are associated with high morbidity and mortality [160]. Gallidermin is a lantibiotic produced by *Staphylococcus gallinarum* and active against various GPB, including MDR species (Table 1) [127]. The effect of gallidermin on clinical isolates of *S. aureus* and *S. epidermidis* grown in planktonic and biofilm modes was assessed [127]. In addition to its effect against planktonic isolates, gallidermin interestingly reduced biofilm formation by both species and exhibited an antibacterial effect against preforming biofilm cells. While concentrations up to 3–4-fold of MIC values were to be used in order to observe a significant effect on cells grown in biofilm, the inhibitory effect of gallidermin on biofilm formation was obtained at sub-lethal concentrations [127]. The authors indicated that, at sub-lethal concentrations, gallidermin inhibited the transcription of the *atl* and *ica* genes necessary for biofilm formation in tested isolates [127]. Similar findings were reported for nisin A and lacticin Q that showed significant antibacterial activity against biofilms formed by a clinical isolate of MRSA [124].

### 5.2. Antibacterial Activity of BGNB

As an example of antimicrobial effect of BGNB, microcins C, L and B17 exhibit the largest antibacterial spectrum, covering *E. coli*, *Shigella* spp., *Salmonella* spp., *Klebsiella* spp. and *Pseudomonas* spp. (Table 1) [152,161,162]. Microcin E492 was reported to exhibit a narrower antibacterial spectrum, including *E. coli*, *Klebsiella pneumoniae* and *Salmonella enteritidis* strains [154]. Unlike the previously mentioned microcins, microcin J25 was noted to have a specific antibacterial activity that is limited to specific *Salmonella* serovars and *E. coli* strains (Table 1) [163,164]. Interestingly, microcin J25 demonstrated the highest activity against pathogenic strains of *E. coli* when compared to other microcins [164]. Similarly, microcin V was noticed to have a potent activity against *E. coli*, while other GNB were resistant to this microcin (Table 1) [62]. Although no antibacterial activity could be related to microcin S, this microcin, isolated from a probiotic *E. coli* strain, was able to inhibit the adherence of enteropathogenic *E. coli* in human intestinal epithelial cellular model, highlighting its role in the competition among these co-occurring strains [165]. Since colicins are generally marked by their specific effect against sensitive strains of the same species, efforts had been made to evaluate their activity against pathogenic and MDR *E. coli* strains [166]. In this context, colicins E1, E6, E7, K and M were screened for eventual activity against *E. coli* strains involved in bacteraemia resulting from urinary tract infections [166]. Microcin E7 was reported to be the most active, since only 13% of the 103 tested strains in this study were resistant, while the remaining microcins were less active, with 32%-53% of tested strains being resistant [166]. Some R-type pyocins were also reported to be particularly active against GNB, other than *P. aeruginosa*, such as *Neisseria gonorrhoeae*, *Neisseria meningitidis*, *Haemophilus ducreyi*, and *Haemophilus influenzae* [61]. As for BGNP, the use of bacteriocins produced by GPB associated to other drugs is an interesting lead, such as the association of microcin J25 and the membrane-permeabilizing peptide (KFF)3K, in order to inhibit *Salmonella enterica* serovar Typhimurium [167].

## 6. Conclusions

Microbial resistance to various antimicrobial agents is increasingly reported and dangerously limits the available therapeutic options. In this context, numerous research works investigate natural resources with the aim to retrieve new antibiotics or alternative therapeutic approach. Consequently, bacteriocins attracted attention for their potential antimicrobial activity. Although several bacteriocins are increasingly reported, many are evaluated against indicator bacterial strains in an attempt to demonstrate their antibacterial activity. However, the real potential of bacteriocins as anti-infective agents would be more accurately appreciated by testing their activity against MDR strains. Indeed, these strains may exhibit a modified membrane structure and composition in order to limit the antibiotic effect [168]. As bacteriocins also must interact with bacterial membrane in order to exhibit their antibacterial activity, these mechanisms of resistance may impact bacteriocin efficiency, highlighting the need to test their activities against MDR isolates more regularly [125,169]. 

Thanks to several studies, some bacteriocins demonstrate veritable potential for further development as antimicrobial agents. It can be noticed that BGPBs represent the major part of bacteriocins reported to date, compared to BGNP. At first glance, it may be thought that BGNP are less attractive antibacterial agents than BGPB, due to their strain-specific antibacterial activity. BGNP may, however, represent an interesting advantage thanks to such a specific antibacterial spectrum. Many GNBs currently present a real threat due to their MDR characteristics such as enterobacterial species and non-fermenting GNB (*P. aeruginosa*, *Acinetobacter baumannii*, *Burkholderia cepacia,* etc.) [170]. Consequently, an interesting approach to develop new antibacterial agents against these bacteria would be to exploit the antibacterial activity of compounds involved in competition among different strains of these species, for example anti-*Pseudomonas* or anti-*Burkholderia* peptides [171,172]. In order to go further, bioengineering technology demonstrated that a great enhancement of bacteriocin antibacterial potential can be achieved, as exemplified by nisin and mersacidin variants [35]. Consequently, it would be pivotal to gather enough information about bacteriocin biology, including biosynthesis machinery, self-immunity and transport, their antibacterial activity, and the underlying mechanisms of action and resistance. The association of drugs and bacteriocin is another way to enhance the antimicrobial effects of these molecules and inhibit MDR strains [158,159].

In addition to assay bacteriocins against MDR and/or pathogenic strains, it is essential to evaluate the toxic effect of new interesting bacteriocins against eukaryotic cells. As compared to reports describing newly discovered bacteriocins, data dealing with bacteriocin cytotoxicity may be limited for certain peptides, highlighting the need to address this important issue that would allow for the early evaluation of bacteriocin antimicrobial potential and safety [173,174,175].

However, it should not be forgotten that continuous exposure to bacteriocins can select resistant bacteria, as is the case for conventional antibacterials. The mechanisms generally found are: (i) reduction in or loss of bacteriocin binding or insertion, (ii) bacteriocin sequestering, (iii) bacteriocin efflux pumping and (iv) bacteriocin degradation, and correspond to changes in the bacterial cell wall [106]. Moreover, it should be noted that other mechanisms of resistance to bacteriocins, involving other proteins and enzymes, have been described in the literature. For example, we can quote the nisinase isolated from several *Bacillus* sp., *Lactobacillus plantarum*, *Streptococcus thermophilus*, *C. botulinum*, *L. lactis* subsp. *cremoris*, *E. faecalis*, and *S. aureus* (see Paragraph 4.3), the nisin resistance protein in *L. lactis*, the glutamate decarboxylase (GAD) system in *L. monocytogenes*, the arginine deiminase pathway (arc operon) in *L. lactis*, the tellurite resistance gene, *telA* in *L. monocytogenes*, etc. [176]. Deciphering such mechanisms of resistance is important, because they can explain why some bacteriocins show limited activity in specific organisms, and this can be very helpful for the design of new antibacterial agents targeting MDR bacteria.

In conclusion, bacteriocins are a promising group of antimicrobial peptides that may present potential alternatives to classical antibiotics in the struggle against antimicrobial resistance. Although numerous bacteriocins are reported, many remain currently undiscovered due to the huge diversity of their natural sources, inviting further research works to be realized in this field. The combination of bacteriocin and antibiotics represent a very interesting possibility to both enhance antimicrobial activity and open new therapeutic possibilities. Thus, characterization of the structure of newly discovered bacteriocins, as well as their biosynthesis, self- immunity, transport mechanisms and mechanisms of action, is pivotal, especially against MDR and/or pathogenic GNB.

## Figures and Tables

**Table 1:** Antibacterial activities of various bacteriocins produced by Gram-positive bacteria (GPB) and Gram-negative bacteria (GNB).

	Bacteriocin	Producer Strain	Sensitive Strains	Antibacterial Activity		Reference
				MIC (mg/L)	Inhibition Diameter (mm)	
Bacteriocins produced by GPB	Nisin A	*Lactococcus lactis*	Methicillin-resistant *Staphylococcus aureus* (MRSA)	0.5–4.1		[124,125]
			Vancomycin-intermediate *Staphylococcus aureus* (VISA)	2–>8.3		
			Vancomycin-resistant *Enterococcus* (VRE)	2–>8.3		
	Epidermin	*Staphylococcus epidermidis*	*Staphylococcus aureus*		>14	[126]
			*Streptococcus agalactiae*		>14	
	Gallidermin	*Staphylococcus gallinarum*	*Staphylococcus aureus*	4–8		[127]
			*Streptococcus epidermidis*	4–8		
	Nukacin ISK-1	*Staphylococcus warneri*	MRSA	10–20		[124]
			MRSE	10–20		
	Mersacidin	*Bacillus* sp.	MRSA	1–32		[128,129]
			*Streptococcus pyogenes*	0.5–8		
			*Streptococcus agalactiae*	1–8		
			*Streptococcus pneumoniae*	1–4		
	Subpeptin JM4-B	*Bacillus subtilis*	*Staphylococcus aureus*		15	[130]
			*Streptococcus faecalis*		25	
			*Salmonella* sp.		22	
			*Shigella flexneri*		15	
	Subtilosin A	*Bacillus subtilis*	*Enterococcus faecalis*	3.125		[131]
			*Listeria monocytogenes*	12.5		
	Sublancin	*Bacillus subtilis*	*Staphylococcus aureus*	4.36		[132]
	Bovicin HC5	*Streptococcus bovis*	*Listeria monocytogenes*		>16	[133]
	Microbisporicin	*Microbispora corallina*	MRSA	≤0.13		[84]
			VISA	≤0.13		
			VRE	0.5–2		
			*Streptococcus pyogenes*	≤0.13		
			*Streptococcus pneumoniae*	≤0.13		
			*Clostridium* spp.	≤0.125		
			*Neisseria meningitidis*	0.5		
			*Neisseria gonorrhoeae*	0.25		
	Bottromycin A2	*Streptomyces* *bottropensis*	MRSA	1		[134]
			VRE	0.5		
	Lysostaphin	*Staphylococcus simulans*	MRSA	0.007–0.125		[135]
	Pediocin PA-1	*Pediococcus acidilactici*	*Listeria monocytogenes*	0.0013–0.0062		[136]
	Curvacin A	*Lactobacillus curvatus*	*Listeria monocytogenes*	0.28–0.69		[136]
	Sakacin P	*Lactobacillus sake*	*Listeria monocytogenes*	0.0034–0.0083		[136]
	Enterocin A	*Enterococcus faecium*	*Listeria monocytogenes*	0.0002–0.0011		[136]
	Enterocin E 50-52	*Enterococcus faecium*	*Staphylococcus aureus*	0.2–0.8		[137]
			*Yersinia enterocolitica*	0.156–1.25		
			*Campylobacter jejuni*	0.025–6.4		
	Enterpco, E-760	*Enterococcus* sp.	*Salmonella enterica*	0.2–0.4		
			*Escherichia coli*	0.1–1.6		
			*Yersinia* spp.	0.1–3.2		
			*Campylobacter* spp.	0.05–1.6		
			*Staphylococcus* spp.	1.6		
			*Listeria monocytogenes*	0.1		
	Lactocyclicin Q	*Lactococcus* sp.	*Enterococcus faecium*	0.71		[138]
			*Enterococcus faecalis*	0.26		
			*Enterococcus durans*	0.71		
			*Enterococcus hirae*	0.71		
			*Listeria monocytogenes*	1.03		
	Lacticin Q	*Lactococcus lactis*	MRSA	5		[124]
	Lariatin A	*Rhodococcus iostii*	*Mycobacterium smegmatis*	3.13		[139]
	Lariatin B	*Rhodococcus iostii*	*Mycobacterium smegmatis*	6.25		[139]
			*Mycobacterium tuberculosis*	0.39		
	Lacticin 3147	*Lactococcus lactis*	MRSA	1.9–15.4		[125,140]
			VRE	1.9–7.7		
			*Mycobacterium tuberculosis*	7.5		
			*Mycobacterium kansasii*	60		
			*Mycobacterium avium*	15		
	Lactocin MXJ 32A	*Lactobacillus coryniformis*	*Staphylococcus aureus*	10		[141]
			*Escherichia coli*	10		
	BMP11	*Lactobacillus crustorum*	*Staphylococcus aureus*	0.3–0.6		[142]
			*Listeria monocytogenes*	0.6		
			*Escherichia coli*	2.4		
			*Salmonella* sp.	0.6		
	L-1077	*Lactobacillus salivarius*	*Salmonella* spp.	0.19–0.38		[143]
			*Escherichia coli*	0.19		
			*Yersinia enterocolitica*	0.76		
			*Klebsiella pneumoniae*	0.76		
			*Staphylococcus aureus*	0.76		
			*Pseudomonas aeruginosa*	0.38		
			*Listeria monocytogenes*	0.19		
			*Campylobacter jejuni*	0.09		
	Microbisporicin NAI-107	*Microbispora* sp.	MRSA	0.06–0.125		[144]
			VRE	0.5–1		
			Penicillin-intermediate *Streptococcus pneumoniae* (PISP)	0.015		
	Mutacin B-Ny266	*Streptococcus mutans*	MRSA	2		[145]
			VRE (*Enterococcus faecalis)*	2.7		
			*Escherichia coli*	1.7		
	KT11	*Enterococcus faecalis*	MRSE		20	[146]
			Methicillin-vancomycin-resistant *Staphylococcus warneri*		20	
			VRE		17	
	Thiazomycin	*Amycolatopsis fastidiosa*	MRSA	0.02–0.1		[147]
			VRE	0.004–0.1		
			*Streptococcus* spp.	0.004–0.03		
	Philipimycin	*Actinoplanes philippinensis*	MRSA	0.125		[148]
			VRE (*Enterococcus faecium*)	0.03		
	Paracaseicin A	*Lactobacillus paracasei*	*Escherichia coli*		18–22	[149]
			*Klebsiella pneumoniae*		16–18	
			MRSA		16–20	
	PJ4	*Lacobacillus helveticus*	*Escherichia coli*		27 ± 0.45	[150]
			*Pseudomonas aeruginosa*		17 ± 0.35	
			*Staphylococcus aureus*		25 ± 0.32	
			*Enterococcus faecalis*		28 ± 0.18	
			*Enterococcus faecium*		28 ± 0.30	
			*Klebsiella pneumoniae*		21 ± 0.27	
			*Salmonella Typhimurium*		17 ± 0.15	
			*Shigella flexneri*		18 ± 0.16	
	VJ13	*Pediococcus pentosaceus*	*Listeria monocytogenes*		27 ± 3	[151]
			*Staphylococcus aureus*		22 ± 2	
			*Bacillus cereus*		22 ± 3	
			*Klebsiella pneumoniae*		20 ± 3	
			*Clostridium sporogenes*		18 ± 3	
			*Citrobacter freundii*		18 ± 2	
			*Proteus vulgaris*		17 ± 3	
			*Clostridium perfringens*		16 ± 3	
			*Streptococcus pyogenes*		16 ± 4	
			*Vibrio parahemolyticus*		16 ± 2	
			*Pseudomonas aureginosa*		15 ± 3	
			*Staphylococcus epidermidis*		15 ± 2	
			*Mycobacterium smegmatis*		14 ± 4	
			*Escherichia coli*		13 ± 2	
Bacteriocins produced by GNB	Microcin L	*Escherichia coli*	*Escherichia coli*		12–18	[152]
			*Salmonella enterica*		12–18	
			*Shigella* spp.		12–18	
			*Pseudomonas aeruginosa*		8–12	
	Microcin J25	*Escherichia coli*	*Escherichia coli*	10.6		[153]
			*Salmonella enterica*	3.2–4.25		
	Microcin E492	*Klebsiella pneumoniae*	*Escherichia coli*	2.37		[154]
			*Salmonella enteritidis*	9.86		

GPB: Gram-positive bacteria; GNB: Gram-negative bacteria; MIC: Minimum inhibitory concentration; MRSE: Methicillin-resistant *Staphylococcus epidermidis*; MRSA: Methicillin-resistant *Staphylococcus aureus*; PISP: Penicillin-intermediate *Streptococcus pneumoniae;* VISA: Vancomycin-intermediate *Staphylococcus aureus*; VRE: Vancomycin-resistant *Enterococcus*.
